# Supplementary material for: Molecular epidemiology and whole genome sequencing analysis of clinical Mycobacterium bovis from Ghana
Source: PLoS One. 2019 Mar 4;14(3):e0209395. doi: 10.1371/journal.pone.0209395 (PMC6398925; doi:10.1371/journal.pone.0209395)
Supplement: S1 Table — (DOCX) [file pone.0209395.s001.docx]

**Supplementary table S1: Global compilation of published *M. bovis* genomes**

| **ENA accession** | **Host** | **Country** | **Year** | **Pubmed ID** |
| --- | --- | --- | --- | --- |
| ERR125598 | Cow | UK | 2004 | 23209404 |
| ERR125599 | Cow | UK | 1999 | 23209404 |
| ERR125600 | Badger | UK | 2006 | 23209404 |
| ERR125601 | Cow | UK | 1999 | 23209404 |
| ERR125602 | Badger | UK | 2005 | 23209404 |
| ERR125603 | Badger | UK | 2005 | 23209404 |
| ERR125604 | Badger | UK | 2007 | 23209404 |
| ERR125605 | Cow | UK | 2003 | 23209404 |
| ERR125606 | Cow | UK | 2004 | 23209404 |
| ERR125607 | Cow | UK | 1999 | 23209404 |
| ERR125608 | Badger | UK | 2004 | 23209404 |
| ERR125609 | Cow | UK | 2008 | 23209404 |
| ERR125610 | Cow | UK | 2010 | 23209404 |
| ERR125611 | Cow | UK | 2007 | 23209404 |
| ERR125612 | Cow | UK | 2008 | 23209404 |
| ERR125613 | Cow | UK | 2007 | 23209404 |
| ERR125614 | Cow | UK | 2007 | 23209404 |
| ERR125615 | Cow | UK | 2008 | 23209404 |
| ERR125616 | Cow | UK | 2010 | 23209404 |
| ERR125617 | Cow | UK | 2008 | 23209404 |
| ERR125618 | Cow | UK | 2007 | 23209404 |
| ERR125619 | Cow | UK | 2010 | 23209404 |
| ERR125620 | Cow | UK | 2010 | 23209404 |
| ERR125621 | Cow | UK | 2010 | 23209404 |
| ERR125622 | Cow | UK | 2010 | 23209404 |
| ERR125623 | Cow | UK | 2010 | 23209404 |
| ERR125624 | Cow | UK | 2010 | 23209404 |
| ERR125625 | Cow | UK | 2010 | 23209404 |
| ERR125626 | Cow | UK | 2010 | 23209404 |
| ERR125627 | Cow | UK | 2010 | 23209404 |
| ERR125628 | Cow | UK | 2010 | 23209404 |
| SRR1657056 | Cow | Guatemala | 2013 | 25988479 |
| SRR1657057 | Cow | Guatemala | 2013 | 25988479 |
| SRR1657061 | Cow | Costa Rica | 2013 | 25988479 |
| SRR1657063 | Cow | Costa Rica | 2013 | 25988479 |
| SRR1657065 | Cow | Guatemala | 2013 | 25988479 |
| SRR1657068 | Cow | Costa Rica | 2013 | 25988479 |
| SRR1657070 | Cow | Panama | 1997 | 25988479 |
| SRR5382721 | Cow | Uruguay | 2014 | 26543108 |
| SRR1791935 | Cow | USA | 2005 | 26785113 |
| SRR1791947 | Cow | USA | 2005 | 26785113 |
| SRR1791967 | Cow | USA | 2005 | 26785113 |
| SRR1791979 | Cow | USA | 2005 | 26785113 |
| SRR1791980 | Cow | USA | 2005 | 26785113 |
| SRR1791981 | Cow | USA | 2005 | 26785113 |
| SRR1791982 | Cow | USA | 2005 | 26785113 |
| SRR1791983 | Cow | USA | 2005 | 26785113 |
| SRR1791984 | Cow | USA | 2005 | 26785113 |
| SRR1791985 | Cow | USA | 2005 | 26785113 |
| SRR1791994 | Cow | USA | 2006 | 26785113 |
| SRR1791995 | White-tailed Deer | USA | 2006 | 26785113 |
| SRR1791996 | Cow | USA | 2006 | 26785113 |
| SRR1791997 | Cow | USA | 2006 | 26785113 |
| SRR1792006 | Cow | USA | 2006 | 26785113 |
| SRR1792007 | Cow | USA | 2006 | 26785113 |
| SRR1792009 | White-tailed Deer | USA | 2006 | 26785113 |
| SRR1792010 | White-tailed Deer | USA | 2006 | 26785113 |
| SRR1792038 | Cow | USA | 2007 | 26785113 |
| SRR1792043 | White-tailed Deer | USA | 2007 | 26785113 |
| SRR1792044 | White-tailed Deer | USA | 2007 | 26785113 |
| SRR1792048 | White-tailed Deer | USA | 2007 | 26785113 |
| SRR1792049 | White-tailed Deer | USA | 2007 | 26785113 |
| SRR1792061 | White-tailed Deer | USA | 2007 | 26785113 |
| SRR1792062 | White-tailed Deer | USA | 2007 | 26785113 |
| SRR1792067 | White-tailed Deer | USA | 2007 | 26785113 |
| SRR1792070 | White-tailed Deer | USA | 2007 | 26785113 |
| SRR1792076 | White-tailed Deer | USA | 2007 | 26785113 |
| SRR1792090 | Cow | USA | 2008 | 26785113 |
| SRR1792094 | White-tailed Deer | USA | 2008 | 26785113 |
| SRR1792095 | White-tailed Deer | USA | 2008 | 26785113 |
| SRR1792097 | White-tailed Deer | USA | 2008 | 26785113 |
| SRR1792100 | Cow | USA | 2008 | 26785113 |
| SRR1792102 | White-tailed Deer | USA | 2008 | 26785113 |
| SRR1792103 | White-tailed Deer | USA | 2008 | 26785113 |
| SRR1792104 | White-tailed Deer | USA | 2008 | 26785113 |
| SRR1792105 | Cow | USA | 2008 | 26785113 |
| SRR1792114 | Cow | USA | 2008 | 26785113 |
| SRR1792115 | Cow | USA | 2008 | 26785113 |
| SRR1792116 | Cow | USA | 2008 | 26785113 |
| SRR1792119 | White-tailed Deer | USA | 2008 | 26785113 |
| SRR1792121 | Cow | USA | 2008 | 26785113 |
| SRR1792123 | White-tailed Deer | USA | 2008 | 26785113 |
| SRR1792124 | White-tailed Deer | USA | 2008 | 26785113 |
| SRR1792142 | White-tailed Deer | USA | 2008 | 26785113 |
| SRR1792143 | White-tailed Deer | USA | 2008 | 26785113 |
| SRR1792144 | White-tailed Deer | USA | 2008 | 26785113 |
| SRR1792145 | White-tailed Deer | USA | 2008 | 26785113 |
| SRR1792149 | White-tailed Deer | USA | 2008 | 26785113 |
| SRR1792166 | Cow | USA | 2009 | 26785113 |
| SRR1792167 | Cow | USA | 2009 | 26785113 |
| SRR1792168 | Cow | USA | 2009 | 26785113 |
| SRR1792177 | Cow | USA | 2009 | 26785113 |
| SRR1792178 | Cow | USA | 2009 | 26785113 |
| SRR1792179 | Cow | USA | 2009 | 26785113 |
| SRR1792180 | Cow | USA | 2009 | 26785113 |
| SRR1792181 | Cow | USA | 2009 | 26785113 |
| SRR1792182 | Cow | USA | 2009 | 26785113 |
| SRR1792183 | Cow | USA | 2009 | 26785113 |
| SRR1792191 | White-tailed Deer | USA | 2009 | 26785113 |
| SRR1792192 | White-tailed Deer | USA | 2009 | 26785113 |
| SRR1792199 | White-tailed Deer | USA | 2009 | 26785113 |
| SRR1792231 | White-tailed Deer | USA | 2009 | 26785113 |
| SRR1792244 | White-tailed Deer | USA | 2010 | 26785113 |
| SRR1792334 | Cow | USA | 2012 | 26785113 |
| SRR1792338 | Cow | USA | 2012 | 26785113 |
| SRR1792339 | Cow | USA | 2012 | 26785113 |
| SRR1792340 | Cow | USA | 2012 | 26785113 |
| SRR1792341 | Cow | USA | 2012 | 26785113 |
| SRR1792429 | Cow | USA | 2013 | 26785113 |
| ERR841787 | Cow | UK | 2008 | 26972511 |
| ERR841788 | Cow | UK | 1998 | 26972511 |
| ERR841789 | Cow | UK | 2004 | 26972511 |
| ERR841790 | Cow | UK | 2005 | 26972511 |
| ERR841791 | Cow | UK | 2004 | 26972511 |
| ERR841792 | Cow | UK | 2004 | 26972511 |
| ERR841793 | Cow | UK | 2000 | 26972511 |
| ERR841794 | Cow | UK | 2000 | 26972511 |
| ERR841795 | Cow | UK | 1999 | 26972511 |
| ERR841796 | Cow | UK | 2000 | 26972511 |
| ERR841797 | Cow | UK | 2003 | 26972511 |
| ERR841798 | Cow | UK | 2003 | 26972511 |
| ERR841799 | Cow | UK | 2004 | 26972511 |
| ERR841800 | Cow | UK | 2003 | 26972511 |
| ERR841801 | Cow | UK | 2003 | 26972511 |
| ERR841802 | Cow | UK | 2005 | 26972511 |
| ERR841803 | Cow | UK | 2003 | 26972511 |
| ERR841804 | Cow | UK | 2005 | 26972511 |
| ERR841805 | Cow | UK | 2003 | 26972511 |
| ERR841806 | Cow | UK | 1998 | 26972511 |
| ERR841807 | Cow | UK | 2005 | 26972511 |
| ERR841808 | Cow | UK | 2004 | 26972511 |
| ERR841809 | Badger | UK | 2007 | 26972511 |
| ERR841810 | Cow | UK | 2005 | 26972511 |
| ERR841811 | Cow | UK | 2009 | 26972511 |
| ERR841812 | Cow | UK | 2006 | 26972511 |
| ERR841813 | Cow | UK | 2007 | 26972511 |
| ERR841814 | Cow | UK | 2009 | 26972511 |
| ERR841815 | Cow | UK | 2008 | 26972511 |
| ERR841816 | Cow | UK | 2007 | 26972511 |
| ERR841817 | Cow | UK | 2006 | 26972511 |
| ERR841818 | Cow | UK | 2006 | 26972511 |
| ERR841819 | Cow | UK | 2006 | 26972511 |
| ERR841820 | Cow | UK | 2006 | 26972511 |
| ERR841821 | Cow | UK | 2007 | 26972511 |
| ERR841822 | Cow | UK | 2006 | 26972511 |
| ERR841823 | Cow | UK | 2008 | 26972511 |
| ERR841824 | Cow | UK | 2006 | 26972511 |
| ERR841825 | Cow | UK | 2007 | 26972511 |
| ERR841826 | Cow | UK | 2009 | 26972511 |
| ERR841827 | Cow | UK | 2009 | 26972511 |
| ERR841828 | Cow | UK | 2006 | 26972511 |
| ERR841829 | Cow | UK | 2006 | 26972511 |
| ERR841830 | Cow | UK | 2009 | 26972511 |
| ERR841831 | Cow | UK | 2008 | 26972511 |
| ERR841832 | Cow | UK | 2006 | 26972511 |
| ERR841833 | Cow | UK | 2009 | 26972511 |
| ERR841834 | Cow | UK | 2010 | 26972511 |
| ERR841835 | Cow | UK | 2010 | 26972511 |
| ERR841836 | Cow | UK | 2010 | 26972511 |
| ERR841837 | Cow | UK | 2010 | 26972511 |
| ERR841838 | Cow | UK | 2010 | 26972511 |
| ERR841839 | Cow | UK | 2010 | 26972511 |
| ERR841840 | Cow | UK | 2010 | 26972511 |
| ERR841841 | Cow | UK | 2010 | 26972511 |
| ERR841842 | Cow | UK | 2010 | 26972511 |
| ERR841843 | Cow | UK | 2010 | 26972511 |
| ERR841844 | Cow | UK | 2010 | 26972511 |
| ERR841845 | Cow | UK | 2010 | 26972511 |
| ERR841846 | Cow | UK | 2010 | 26972511 |
| ERR841847 | Cow | UK | 2010 | 26972511 |
| ERR841848 | Cow | UK | 2010 | 26972511 |
| ERR841849 | Cow | UK | 2010 | 26972511 |
| ERR841850 | Cow | UK | 2010 | 26972511 |
| ERR841851 | Cow | UK | 2010 | 26972511 |
| ERR841852 | Cow | UK | 2010 | 26972511 |
| ERR841853 | Cow | UK | 2010 | 26972511 |
| ERR841854 | Cow | UK | 2010 | 26972511 |
| ERR841855 | Cow | UK | 2010 | 26972511 |
| ERR841856 | Cow | UK | 2010 | 26972511 |
| ERR841857 | Cow | UK | 2011 | 26972511 |
| ERR841858 | Cow | UK | 2010 | 26972511 |
| ERR841859 | Cow | UK | 2011 | 26972511 |
| ERR841860 | Cow | UK | 2011 | 26972511 |
| ERR841861 | Cow | UK | 2011 | 26972511 |
| ERR841862 | Cow | UK | 2011 | 26972511 |
| ERR841863 | Cow | UK | 2011 | 26972511 |
| ERR841864 | Cow | UK | 2011 | 26972511 |
| ERR841865 | Cow | UK | 2010 | 26972511 |
| ERR841867 | Cow | UK | 2011 | 26972511 |
| ERR841868 | Cow | UK | 2011 | 26972511 |
| ERR841869 | Cow | UK | 2011 | 26972511 |
| ERR841870 | Cow | UK | 2011 | 26972511 |
| ERR841871 | Cow | UK | 2011 | 26972511 |
| ERR841872 | Cow | UK | 2011 | 26972511 |
| ERR841873 | Cow | UK | 2011 | 26972511 |
| ERR841874 | Cow | UK | 2011 | 26972511 |
| ERR841875 | Cow | UK | 2011 | 26972511 |
| ERR841877 | Cow | UK | 2011 | 26972511 |
| ERR841878 | Cow | UK | 2011 | 26972511 |
| ERR841879 | Cow | UK | 2010 | 26972511 |
| ERR841880 | Cow | UK | 2011 | 26972511 |
| ERR841881 | Cow | UK | 2011 | 26972511 |
| ERR841882 | Cow | UK | 2010 | 26972511 |
| ERR841883 | Cow | UK | 2011 | 26972511 |
| ERR841884 | Cow | UK | 2011 | 26972511 |
| ERR841885 | Cow | UK | 2007 | 26972511 |
| ERR841886 | Cow | UK | 2003 | 26972511 |
| ERR841887 | Cow | UK | 2003 | 26972511 |
| ERR841888 | Cow | UK | 2002 | 26972511 |
| ERR841889 | Cow | UK | 2002 | 26972511 |
| ERR841890 | Cow | UK | 2005 | 26972511 |
| ERR841891 | Cow | UK | 2004 | 26972511 |
| ERR841892 | Cow | UK | 2011 | 26972511 |
| ERR841893 | Cow | UK | 2004 | 26972511 |
| ERR841894 | Cow | UK | 2004 | 26972511 |
| ERR841895 | Cow | UK | 2011 | 26972511 |
| ERR841896 | Cow | UK | 2005 | 26972511 |
| ERR841897 | Cow | UK | 2003 | 26972511 |
| ERR841898 | Cow | UK | 2003 | 26972511 |
| ERR841899 | Cow | UK | 1998 | 26972511 |
| ERR841900 | Cow | UK | 2011 | 26972511 |
| ERR841901 | Cow | UK | 2011 | 26972511 |
| ERR841902 | Cow | UK | 2011 | 26972511 |
| SRR4117155 | Wild boar | France | 2010 | 27834714 |
| ERR564260 | Possum | New Zealand | 1988 | 28209138 |
| ERR564261 | Cow | New Zealand | 1993 | 28209138 |
| ERR564262 | Deer | New Zealand | 1994 | 28209138 |
| ERR564263 | Cow | New Zealand | 1994 | 28209138 |
| ERR564264 | Cow | New Zealand | 1995 | 28209138 |
| ERR564265 | Possum | New Zealand | 2000 | 28209138 |
| ERR564266 | Cow | New Zealand | 2001 | 28209138 |
| ERR564267 | Cow | New Zealand | 2001 | 28209138 |
| ERR564268 | Deer | New Zealand | 1998 | 28209138 |
| ERR564269 | Cow | New Zealand | 2002 | 28209138 |
| ERR564270 | Cow | New Zealand | 1996 | 28209138 |
| ERR564271 | Possum | New Zealand | 1997 | 28209138 |
| ERR564272 | Cow | New Zealand | 1998 | 28209138 |
| ERR564273 | Cow | New Zealand | 1998 | 28209138 |
| ERR564274 | Cow | New Zealand | 1998 | 28209138 |
| ERR564275 | Cow | New Zealand | 1998 | 28209138 |
| ERR564276 | Cow | New Zealand | 2003 | 28209138 |
| ERR564277 | Ferret | New Zealand | 2004 | 28209138 |
| ERR564278 | Pig | New Zealand | 2004 | 28209138 |
| ERR564279 | Cow | New Zealand | 2006 | 28209138 |
| ERR564280 | Cow | New Zealand | 2002 | 28209138 |
| ERR564281 | Cow | New Zealand | 2002 | 28209138 |
| ERR564282 | Cow | New Zealand | 2005 | 28209138 |
| ERR564283 | Cow | New Zealand | 1992 | 28209138 |
| ERR564284 | Cow | New Zealand | 1998 | 28209138 |
| ERR564285 | Possum | New Zealand | 1983 | 28209138 |
| ERR564286 | Cow | New Zealand | 1984 | 28209138 |
| ERR564287 | Cow | New Zealand | 2002 | 28209138 |
| ERR564288 | Cow | New Zealand | 2002 | 28209138 |
| ERR564289 | Cow | New Zealand | 1984 | 28209138 |
| ERR564290 | Cow | New Zealand | 1994 | 28209138 |
| ERR564291 | Deer | New Zealand | 2003 | 28209138 |
| ERR564292 | Cow | New Zealand | 2002 | 28209138 |
| ERR564293 | Ferret | New Zealand | 1998 | 28209138 |
| ERR564294 | Ferret | New Zealand | 2003 | 28209138 |
| ERR564295 | Cow | New Zealand | 2002 | 28209138 |
| ERR564296 | Cow | New Zealand | 1999 | 28209138 |
| ERR564297 | Cow | New Zealand | 2001 | 28209138 |
| ERR564298 | Cow | New Zealand | 2002 | 28209138 |
| ERR564299 | Cow | New Zealand | 1992 | 28209138 |
| ERR564300 | Cow | New Zealand | 2008 | 28209138 |
| ERR564301 | Possum | New Zealand | 1988 | 28209138 |
| ERR564302 | Cow | New Zealand | 2006 | 28209138 |
| ERR564303 | Cow | New Zealand | 2001 | 28209138 |
| ERR564304 | Cow | New Zealand | 1996 | 28209138 |
| ERR564305 | Cow | New Zealand | 2001 | 28209138 |
| ERR564306 | Ferret | New Zealand | 1999 | 28209138 |
| ERR564307 | Possum | New Zealand | 2000 | 28209138 |
| ERR564308 | Possum | New Zealand | 1992 | 28209138 |
| ERR564309 | Ferret | New Zealand | 2004 | 28209138 |
| ERR564310 | Ferret | New Zealand | 1993 | 28209138 |
| ERR564311 | Ferret | New Zealand | 1996 | 28209138 |
| ERR564312 | Cow | New Zealand | 1994 | 28209138 |
| ERR564313 | Cow | New Zealand | 2005 | 28209138 |
| ERR564314 | Deer | New Zealand | 1994 | 28209138 |
| ERR564315 | Ferret | New Zealand | 1996 | 28209138 |
| ERR564316 | Cow | New Zealand | 1996 | 28209138 |
| ERR564317 | Cow | New Zealand | 1995 | 28209138 |
| ERR564318 | Deer | New Zealand | 2001 | 28209138 |
| ERR564319 | Cow | New Zealand | 2002 | 28209138 |
| ERR564320 | Cow | New Zealand | 1999 | 28209138 |
| ERR564321 | Cow | New Zealand | 1999 | 28209138 |
| ERR564322 | Cow | New Zealand | 2000 | 28209138 |
| ERR564323 | Cow | New Zealand | 2002 | 28209138 |
| ERR564324 | Ferret | New Zealand | 2003 | 28209138 |
| ERR564325 | Possum | New Zealand | 1999 | 28209138 |
| ERR564326 | Cow | New Zealand | 2000 | 28209138 |
| ERR564327 | Possum | New Zealand | 1998 | 28209138 |
| ERR564328 | Cow | New Zealand | 2007 | 28209138 |
| ERR564329 | Cow | New Zealand | 1997 | 28209138 |
| ERR564330 | Cow | New Zealand | 1997 | 28209138 |
| ERR564331 | Possum | New Zealand | 1985 | 28209138 |
| ERR564332 | Ferret | New Zealand | 1997 | 28209138 |
| ERR564333 | Ferret | New Zealand | 1992 | 28209138 |
| ERR564334 | Cow | New Zealand | 1987 | 28209138 |
| ERR564335 | Cow | New Zealand | 1997 | 28209138 |
| ERR564336 | Cow | New Zealand | 1992 | 28209138 |
| ERR564337 | Cow | New Zealand | 1998 | 28209138 |
| ERR564338 | Deer | New Zealand | 1999 | 28209138 |
| ERR564339 | Cow | New Zealand | 2008 | 28209138 |
| ERR564340 | Cow | New Zealand | 2005 | 28209138 |
| ERR564341 | Cow | New Zealand | 1998 | 28209138 |
| ERR564342 | Possum | New Zealand | 1985 | 28209138 |
| ERR564343 | Cow | New Zealand | 1999 | 28209138 |
| ERR564344 | Cow | New Zealand | 1998 | 28209138 |
| ERR564345 | Cow | New Zealand | 2005 | 28209138 |
| ERR564346 | Cow | New Zealand | 1997 | 28209138 |
| ERR564347 | Ferret | New Zealand | 2007 | 28209138 |
| ERR564348 | Cow | New Zealand | 2006 | 28209138 |
| ERR564349 | Ferret | New Zealand | 1992 | 28209138 |
| ERR564350 | Cow | New Zealand | 1999 | 28209138 |
| ERR564351 | Cow | New Zealand | 1996 | 28209138 |
| ERR564352 | Possum | New Zealand | 1997 | 28209138 |
| ERR564353 | Deer | New Zealand | 1997 | 28209138 |
| ERR564354 | Possum | New Zealand | 1999 | 28209138 |
| ERR564355 | Cow | New Zealand | 2007 | 28209138 |
| ERR564356 | Cow | New Zealand | 2008 | 28209138 |
| ERR564357 | Ferret | New Zealand | 2006 | 28209138 |
| ERR564358 | Possum | New Zealand | 1992 | 28209138 |
| ERR564359 | Cow | New Zealand | 2006 | 28209138 |
| ERR564360 | Cow | New Zealand | 2008 | 28209138 |
| ERR564361 | Deer | New Zealand | 1992 | 28209138 |
| ERR564362 | Possum | New Zealand | 1985 | 28209138 |
| ERR564363 | Ferret | New Zealand | 2001 | 28209138 |
| ERR564364 | Ferret | New Zealand | 1993 | 28209138 |
| ERR564365 | Possum | New Zealand | 1988 | 28209138 |
| ERR564366 | Cow | New Zealand | 2000 | 28209138 |
| ERR564367 | Cow | New Zealand | 1993 | 28209138 |
| ERR564368 | Pig | New Zealand | 1992 | 28209138 |
| ERR564369 | Possum | New Zealand | 1993 | 28209138 |
| ERR564370 | Cow | New Zealand | 2001 | 28209138 |
| ERR564371 | Cow | New Zealand | 1992 | 28209138 |
| ERR564372 | Possum | New Zealand | 2001 | 28209138 |
| ERR564373 | Ferret | New Zealand | 2002 | 28209138 |
| ERR564374 | Ferret | New Zealand | 1993 | 28209138 |
| ERR564375 | Cow | New Zealand | 1993 | 28209138 |
| ERR564376 | Possum | New Zealand | 1983 | 28209138 |
| ERR564377 | Cat | New Zealand | 1993 | 28209138 |
| ERR564378 | Pig | New Zealand | 1992 | 28209138 |
| ERR564379 | Possum | New Zealand | 1993 | 28209138 |
| ERR564380 | Pig | New Zealand | 1992 | 28209138 |
| ERR564381 | Pig | New Zealand | 1982 | 28209138 |
| ERR564382 | Cow | New Zealand | 1999 | 28209138 |
| ERR564383 | Possum | New Zealand | 1992 | 28209138 |
| ERR564384 | Cow | New Zealand | 1998 | 28209138 |
| ERR564385 | Pig | New Zealand | 1987 | 28209138 |
| ERR564386 | Cow | New Zealand | 1992 | 28209138 |
| ERR564387 | Cow | New Zealand | 1993 | 28209138 |
| ERR564388 | Cow | New Zealand | 1995 | 28209138 |
| ERR564389 | Ferret | New Zealand | 1998 | 28209138 |
| ERR564390 | Ferret | New Zealand | 1993 | 28209138 |
| ERR564391 | Possum | New Zealand | 2001 | 28209138 |
| ERR564392 | Cow | New Zealand | 1994 | 28209138 |
| ERR564393 | Ferret | New Zealand | 2001 | 28209138 |
| ERR564394 | Cow | New Zealand | 1994 | 28209138 |
| ERR564395 | Cow | New Zealand | 1996 | 28209138 |
| ERR564396 | Cow | New Zealand | 1995 | 28209138 |
| ERR564397 | Ferret | New Zealand | 2002 | 28209138 |
| ERR564398 | Pig | New Zealand | 2001 | 28209138 |
| ERR564399 | Cow | New Zealand | 1997 | 28209138 |
| ERR564400 | Ferret | New Zealand | 1993 | 28209138 |
| ERR564401 | Ferret | New Zealand | 2003 | 28209138 |
| ERR564402 | Cow | New Zealand | 1990 | 28209138 |
| ERR564403 | Cow | New Zealand | 2005 | 28209138 |
| ERR564404 | Cow | New Zealand | 2004 | 28209138 |
| ERR564405 | Cow | New Zealand | 2004 | 28209138 |
| ERR564406 | Cow | New Zealand | 2005 | 28209138 |
| ERR564407 | Cow | New Zealand | 1999 | 28209138 |
| ERR564408 | Possum | New Zealand | 1989 | 28209138 |
| ERR564409 | Cow | New Zealand | 2005 | 28209138 |
| ERR564410 | Ferret | New Zealand | 2002 | 28209138 |
| ERR564411 | Cow | New Zealand | 2005 | 28209138 |
| ERR564412 | Cow | New Zealand | 2002 | 28209138 |
| ERR564413 | Cow | New Zealand | 2005 | 28209138 |
| ERR564414 | Cow | New Zealand | 2002 | 28209138 |
| ERR564415 | Ferret | New Zealand | 2003 | 28209138 |
| ERR564416 | Ferret | New Zealand | 2004 | 28209138 |
| ERR564417 | Cow | New Zealand | 2000 | 28209138 |
| ERR564418 | Ferret | New Zealand | 2008 | 28209138 |
| ERR564419 | Ferret | New Zealand | 2002 | 28209138 |
| ERR564420 | Cow | New Zealand | 2003 | 28209138 |
| ERR564421 | Cow | New Zealand | 1999 | 28209138 |
| ERR564422 | Cow | New Zealand | 1991 | 28209138 |
| ERR564423 | Ferret | New Zealand | 2002 | 28209138 |
| ERR564424 | Ferret | New Zealand | 2005 | 28209138 |
| ERR564425 | Ferret | New Zealand | 1996 | 28209138 |
| ERR564426 | Cow | New Zealand | 2005 | 28209138 |
| ERR564427 | Cow | New Zealand | 2000 | 28209138 |
| ERR564428 | Cow | New Zealand | 2003 | 28209138 |
| ERR564429 | Deer | New Zealand | 2003 | 28209138 |
| ERR564430 | Cow | New Zealand | 1999 | 28209138 |
| ERR564431 | Cow | New Zealand | 2004 | 28209138 |
| ERR564432 | Deer | New Zealand | 2004 | 28209138 |
| ERR564433 | Ferret | New Zealand | 1999 | 28209138 |
| ERR564434 | Cow | New Zealand | 1991 | 28209138 |
| ERR564435 | Cow | New Zealand | 1998 | 28209138 |
| ERR564436 | Stoat | New Zealand | 2005 | 28209138 |
| ERR564437 | Possum | New Zealand | 2001 | 28209138 |
| ERR564438 | Cow | New Zealand | 2004 | 28209138 |
| ERR564439 | Deer | New Zealand | 1993 | 28209138 |
| ERR564440 | Possum | New Zealand | 1994 | 28209138 |
| ERR564441 | Cow | New Zealand | 2005 | 28209138 |
| ERR564442 | Cow | New Zealand | 1993 | 28209138 |
| ERR564443 | Cow | New Zealand | 1999 | 28209138 |
| ERR564444 | Possum | New Zealand | 1996 | 28209138 |
| ERR564445 | Cow | New Zealand | 2001 | 28209138 |
| ERR564446 | Deer | New Zealand | 2004 | 28209138 |
| ERR564447 | Cow | New Zealand | 2004 | 28209138 |
| ERR564448 | Pig | New Zealand | 2001 | 28209138 |
| ERR564449 | Cow | New Zealand | 2004 | 28209138 |
| ERR564450 | Cow | New Zealand | 2007 | 28209138 |
| ERR564451 | Cow | New Zealand | 2007 | 28209138 |
| ERR564452 | Cow | New Zealand | 2010 | 28209138 |
| ERR564453 | Pig | New Zealand | 2006 | 28209138 |
| ERR564454 | Cow | New Zealand | 1998 | 28209138 |
| ERR564455 | Ferret | New Zealand | 2006 | 28209138 |
| ERR564456 | Cow | New Zealand | 2006 | 28209138 |
| ERR564457 | Cow | New Zealand | 2005 | 28209138 |
| ERR564458 | Ferret | New Zealand | 2006 | 28209138 |
| ERR564459 | Cow | New Zealand | 2005 | 28209138 |
| ERR564460 | Cow | New Zealand | 2007 | 28209138 |
| ERR564461 | Cow | New Zealand | 2009 | 28209138 |
| ERR564462 | Cow | New Zealand | 2009 | 28209138 |
| ERR564463 | Cow | New Zealand | 2008 | 28209138 |
| ERR564464 | Possum | New Zealand | 1998 | 28209138 |
| ERR564465 | Cow | New Zealand | 2006 | 28209138 |
| ERR564466 | Cow | New Zealand | 2009 | 28209138 |
| ERR564467 | Cow | New Zealand | 2009 | 28209138 |
| ERR564468 | Pig | New Zealand | 2006 | 28209138 |
| ERR564469 | Cat | New Zealand | 2002 | 28209138 |
| ERR564470 | Cow | New Zealand | 2009 | 28209138 |
| ERR564471 | Cow | New Zealand | 2009 | 28209138 |
| ERR564472 | Cow | New Zealand | 2006 | 28209138 |
| ERR564473 | Cow | New Zealand | 2006 | 28209138 |
| ERR564474 | Cow | New Zealand | 2013 | 28209138 |
| ERR564475 | Cow | New Zealand | 2013 | 28209138 |
| ERR564476 | Cow | New Zealand | 2013 | 28209138 |
| ERR564477 | Cow | New Zealand | 2013 | 28209138 |
| SRR5216690 | Cow | New Zealand | 1998 | 28209138 |
| SRR5216694 | Cow | New Zealand | 2009 | 28209138 |
| SRR5216696 | Possum | New Zealand | 1985 | 28209138 |
| SRR5216701 | Cow | New Zealand | 2013 | 28209138 |
| SRR5216710 | Cow | New Zealand | 2008 | 28209138 |
| SRR5216717 | Cow | New Zealand | 2004 | 28209138 |
| SRR5216726 | Cow | New Zealand | 2007 | 28209138 |
| SRR5216728 | Cow | New Zealand | 2008 | 28209138 |
| SRR5216729 | Possum | New Zealand | 1989 | 28209138 |
| SRR5216730 | Possum | New Zealand | 1992 | 28209138 |
| SRR5216734 | Cow | New Zealand | 2013 | 28209138 |
| SRR5216735 | Cow | New Zealand | 2002 | 28209138 |
| SRR5216737 | Cow | New Zealand | 2010 | 28209138 |
| SRR5216745 | Cow | New Zealand | 2005 | 28209138 |
| SRR5216746 | Possum | New Zealand | 1987 | 28209138 |
| SRR5216749 | Pig | New Zealand | 2004 | 28209138 |
| SRR5216750 | Cow | New Zealand | 2008 | 28209138 |
| SRR5216753 | Possum | New Zealand | 2006 | 28209138 |
| SRR5216756 | Cow | New Zealand | 2005 | 28209138 |
| SRR5216757 | Possum | New Zealand | 2002 | 28209138 |
| SRR5216765 | Deer | New Zealand | 1994 | 28209138 |
| SRR5216766 | Cow | New Zealand | 2013 | 28209138 |
| SRR5216770 | Cow | New Zealand | 1989 | 28209138 |
| SRR5216775 | Cow | New Zealand | 2007 | 28209138 |
| SRR5216776 | Cow | New Zealand | 2005 | 28209138 |
| SRR5216777 | Cow | New Zealand | 2013 | 28209138 |
| SRR5216778 | Cow | New Zealand | 2013 | 28209138 |
| SRR5216780 | Cow | New Zealand | 2009 | 28209138 |
| SRR5216784 | Cow | New Zealand | 2013 | 28209138 |
| SRR5216785 | Cow | New Zealand | 2002 | 28209138 |
| SRR5216789 | Cow | New Zealand | 1991 | 28209138 |
| SRR5216792 | Cow | New Zealand | 2011 | 28209138 |
| SRR5216800 | Cow | New Zealand | 2013 | 28209138 |
| SRR5216803 | Possum | New Zealand | 1992 | 28209138 |
| SRR5216804 | Cow | New Zealand | 2006 | 28209138 |
| SRR5216806 | Ferret | New Zealand | 1997 | 28209138 |
| SRR5216810 | Possum | New Zealand | 2012 | 28209138 |
| SRR5216813 | Cow | New Zealand | 2007 | 28209138 |
| SRR5216815 | Cow | New Zealand | 2010 | 28209138 |
| SRR5216816 | Cow | New Zealand | 2011 | 28209138 |
| SRR5216819 | Cow | New Zealand | 1987 | 28209138 |
| SRR5216822 | Cow | New Zealand | 2006 | 28209138 |
| SRR5216823 | Cow | New Zealand | 2008 | 28209138 |
| SRR5216832 | Cow | New Zealand | 2008 | 28209138 |
| SRR5216843 | Possum | New Zealand | 1999 | 28209138 |
| SRR5216856 | Cow | New Zealand | 2006 | 28209138 |
| SRR5216857 | Cow | New Zealand | 2010 | 28209138 |
| SRR5216858 | Possum | New Zealand | 1999 | 28209138 |
| SRR5216860 | Cow | New Zealand | 2011 | 28209138 |
| SRR5216866 | Cow | New Zealand | 2013 | 28209138 |
| SRR5216867 | Ferret | New Zealand | 1991 | 28209138 |
| SRR5216868 | Cow | New Zealand | 2008 | 28209138 |
| SRR5216869 | Cow | New Zealand | 2008 | 28209138 |
| SRR5216870 | Cow | New Zealand | 2001 | 28209138 |
| SRR5216872 | Cow | New Zealand | 2010 | 28209138 |
| SRR5216873 | Possum | New Zealand | 1999 | 28209138 |
| SRR5216874 | Cow | New Zealand | 2008 | 28209138 |
| SRR5216877 | Possum | New Zealand | 2009 | 28209138 |
| SRR5216879 | Possum | New Zealand | 2005 | 28209138 |
| SRR5216888 | Possum | New Zealand | 1985 | 28209138 |
| SRR5216895 | Possum | New Zealand | 2012 | 28209138 |
| SRR5216896 | Cow | New Zealand | 2005 | 28209138 |
| SRR5216897 | Cow | New Zealand | 2010 | 28209138 |
| SRR5216898 | Possum | New Zealand | 2007 | 28209138 |
| SRR5216900 | Cow | New Zealand | 2001 | 28209138 |
| SRR5216901 | Cow | New Zealand | 2003 | 28209138 |
| SRR5216902 | Cow | New Zealand | 2007 | 28209138 |
| SRR5216905 | Cow | New Zealand | 2008 | 28209138 |
| SRR5216907 | Cow | New Zealand | 1988 | 28209138 |
| SRR5216912 | Possum | New Zealand | 2011 | 28209138 |
| SRR5216915 | Cow | New Zealand | 2009 | 28209138 |
| SRR5216917 | Possum | New Zealand | 1999 | 28209138 |
| SRR5216919 | Possum | New Zealand | 2007 | 28209138 |
| SRR5216920 | Ferret | New Zealand | 1992 | 28209138 |
| SRR5216922 | Possum | New Zealand | 2006 | 28209138 |
| SRR5216924 | Cow | New Zealand | 2011 | 28209138 |
| SRR5216930 | Cow | New Zealand | 2004 | 28209138 |
| SRR5216931 | Cow | New Zealand | 2008 | 28209138 |
| SRR5216932 | Cow | New Zealand | 2004 | 28209138 |
| SRR5216933 | Cow | New Zealand | 2007 | 28209138 |
| SRR5216935 | Cow | New Zealand | 2013 | 28209138 |
| SRR5216942 | Cow | New Zealand | 2005 | 28209138 |
| SRR5216945 | Possum | New Zealand | 1992 | 28209138 |
| SRR5216948 | Cow | New Zealand | 2013 | 28209138 |
| SRR5216951 | Possum | New Zealand | 1984 | 28209138 |
| SRR5216953 | Cow | New Zealand | 1989 | 28209138 |
| SRR5216955 | Cow | New Zealand | 2003 | 28209138 |
| SRR5216960 | Possum | New Zealand | 1982 | 28209138 |
| SRR5216963 | Cow | New Zealand | 2006 | 28209138 |
| SRR5216964 | Possum | New Zealand | 1984 | 28209138 |
| SRR5216968 | Cow | New Zealand | 2010 | 28209138 |
| SRR5216969 | Cow | New Zealand | 2013 | 28209138 |
| SRR5216972 | Possum | New Zealand | 1985 | 28209138 |
| SRR5216973 | Possum | New Zealand | 1983 | 28209138 |
| SRR5216974 | Possum | New Zealand | 1984 | 28209138 |
| SRR5216977 | Cow | New Zealand | 2009 | 28209138 |
| SRR5216984 | Possum | New Zealand | 1983 | 28209138 |
| SRR5216985 | Cow | New Zealand | 2009 | 28209138 |
| ERR1815538 | Lion | South Africa | 1998 | 28412523 |
| ERR1815539 | Buffalo | South Africa | 2000 | 28412523 |
| ERR1815540 | Kudu | South Africa | 1997 | 28412523 |
| ERR1815541 | Buffalo | South Africa | 1999 | 28412523 |
| ERR1815542 | Buffalo | South Africa | 1998 | 28412523 |
| ERR1815543 | Buffalo | South Africa | 1999 | 28412523 |
| ERR1815544 | Cow | South Africa | 2003 | 28412523 |
| ERR1815545 | Buffalo | South Africa | 1998 | 28412523 |
| ERR1815546 | Buffalo | South Africa | 1996 | 28412523 |
| ERR1815547 | Buffalo | South Africa | 2000 | 28412523 |
| ERR1815548 | Cow | South Africa | 2000 | 28412523 |
| ERR1815549 | Lion | South Africa | 1999 | 28412523 |
| ERR1815550 | Lion | South Africa | 1998 | 28412523 |
| SRR5196685 | Wild boar | France | 2011 | 28684564 |
| SRR5657358 | Cow | Mexico | 2015 | 28739421 |
| SRR5657359 | Cow | Mexico | 2015 | 28739421 |
| SRR5657360 | Cow | Mexico | 2015 | 28739421 |
| SRR5657361 | Cow | Mexico | 2015 | 28739421 |
| SRR5657362 | Cow | Mexico | 2015 | 28739421 |
| SRR5657363 | Cow | Mexico | 2015 | 28739421 |
| SRR5657364 | Cow | Mexico | 2015 | 28739421 |
| SRR5657365 | Cow | Mexico | 2015 | 28739421 |
| SRR5657366 | Cow | Mexico | 2015 | 28739421 |
| SRR5657367 | Cow | Mexico | 2015 | 28739421 |
| SRR5657368 | Cow | Mexico | 2015 | 28739421 |
| SRR5657369 | Cow | Mexico | 2015 | 28739421 |
| SRR5657370 | Cow | Mexico | 2015 | 28739421 |
| SRR5657371 | Cow | Mexico | 2015 | 28739421 |
| SRR5657372 | Cow | Mexico | 2015 | 28739421 |
| SRR5657373 | Cow | Mexico | 2015 | 28739421 |
| SRR5657374 | Cow | Mexico | 2015 | 28739421 |
| SRR5657375 | Cow | Mexico | 2015 | 28739421 |
| SRR5657376 | Cow | Mexico | 2015 | 28739421 |
| SRR5657377 | Cow | Mexico | 2015 | 28739421 |
| SRR5657378 | Cow | Mexico | 2015 | 28739421 |
| SRR5657379 | Cow | Mexico | 2015 | 28739421 |
| SRR5657380 | Cow | Mexico | 2015 | 28739421 |
| SRR5657381 | Cow | Mexico | 2015 | 28739421 |
| SRR5657382 | Cow | Mexico | 2015 | 28739421 |
| SRR5657383 | Cow | Mexico | 2015 | 28739421 |
| SRR5657384 | Cow | Mexico | 2015 | 28739421 |
| SRR5657385 | Cow | Mexico | 2015 | 28739421 |
| SRR5657386 | Cow | Mexico | 2015 | 28739421 |
| SRR5657387 | Cow | Mexico | 2015 | 28739421 |
| SRR5657388 | Cow | Mexico | 2015 | 28739421 |
| SRR5657389 | Cow | Mexico | 2015 | 28739421 |
| SRR5657390 | Cow | Mexico | 2015 | 28739421 |
| SRR5657391 | Cow | Mexico | 2015 | 28739421 |
| SRR5657392 | Cow | Mexico | 2015 | 28739421 |
| SRR5657393 | Cow | Mexico | 2015 | 28739421 |
| SRR5657394 | Cow | Mexico | 2015 | 28739421 |
| SRR5657395 | Cow | Mexico | 2015 | 28739421 |
| SRR5657396 | Cow | Mexico | 2015 | 28739421 |
| SRR5657397 | Cow | Mexico | 2015 | 28739421 |
| SRR5657398 | Cow | Mexico | 2015 | 28739421 |
| SRR5657399 | Cow | Mexico | 2015 | 28739421 |
| SRR5657400 | Human | Mexico | 2011 | 28739421 |
| SRR5657401 | Human | Mexico | 2012 | 28739421 |
| SRR5657402 | Cow | Mexico | 2015 | 28739421 |
| SRR5657403 | Cow | Mexico | 2015 | 28739421 |
| SRR5657404 | Cow | Mexico | 2015 | 28739421 |
| SRR5657405 | Cow | Mexico | 2015 | 28739421 |
| SRR5657406 | Cow | Mexico | 2015 | 28739421 |
| SRR5657407 | Cow | Mexico | 2015 | 28739421 |
| SRR5657408 | Human | Mexico | 2011 | 28739421 |
| SRR5657409 | Human | Mexico | 2011 | 28739421 |
| SRR5657410 | Human | Mexico | 2011 | 28739421 |
| SRR5657411 | Cow | Mexico | 2015 | 28739421 |
| SRR5657412 | Cow | Mexico | 2015 | 28739421 |
| SRR5657413 | Cow | Mexico | 2015 | 28739421 |
| SRR5657414 | Cow | Mexico | 2015 | 28739421 |
| SRR5657415 | Cow | Mexico | 2015 | 28739421 |
| SRR5657416 | Cow | Mexico | 2015 | 28739421 |
| SRR5657417 | Cow | Mexico | 2015 | 28739421 |
| SRR5657418 | Cow | Mexico | 2015 | 28739421 |
| SRR5657419 | Cow | Mexico | 2015 | 28739421 |
| SRR5657420 | Cow | Mexico | 2015 | 28739421 |
| SRR5657421 | Cow | Mexico | 2015 | 28739421 |
| SRR5657422 | Cow | Mexico | 2015 | 28739421 |
| SRR5657423 | Cow | Mexico | 2015 | 28739421 |
| SRR5657424 | Cow | Mexico | 2015 | 28739421 |
| SRR5657425 | Cow | Mexico | 2015 | 28739421 |
| SRR5657426 | Cow | Mexico | 2015 | 28739421 |
| SRR5657427 | Cow | Mexico | 2015 | 28739421 |
| SRR5657428 | Cow | Mexico | 2015 | 28739421 |
| SRR5657429 | Cow | Mexico | 2015 | 28739421 |
| SRR5657430 | Cow | Mexico | 2015 | 28739421 |
| SRR5657431 | Cow | Mexico | 2015 | 28739421 |
| SRR5657432 | Cow | Mexico | 2015 | 28739421 |
| SRR5657433 | Cow | Mexico | 2015 | 28739421 |
| SRR5657434 | Cow | Mexico | 2015 | 28739421 |
| SRR5657435 | Cow | Mexico | 2015 | 28739421 |
| SRR5657436 | Cow | Mexico | 2015 | 28739421 |
| SRR5657437 | Cow | Mexico | 2015 | 28739421 |
| SRR5657438 | Cow | Mexico | 2015 | 28739421 |
| SRR5657439 | Cow | Mexico | 2015 | 28739421 |
| SRR5657440 | Cow | Mexico | 2015 | 28739421 |
| SRR5657441 | Cow | Mexico | 2015 | 28739421 |
| SRR5657442 | Cow | Mexico | 2015 | 28739421 |
| SRR5657443 | Cow | Mexico | 2015 | 28739421 |
| SRR5657444 | Cow | Mexico | 2015 | 28739421 |
| SRR5657445 | Cow | Mexico | 2015 | 28739421 |
| SRR5657446 | Cow | Mexico | 2015 | 28739421 |
| SRR5657447 | Cow | Mexico | 2015 | 28739421 |
| SRR5657448 | Cow | Mexico | 2015 | 28739421 |
| SRR5657449 | Cow | Mexico | 2015 | 28739421 |
| SRR5657450 | Cow | Mexico | 2015 | 28739421 |
| SRR5657451 | Cow | Mexico | 2015 | 28739421 |
| SRR5657452 | Cow | Mexico | 2015 | 28739421 |
| SRR5657453 | Cow | Mexico | 2015 | 28739421 |
| SRR5657454 | Cow | Mexico | 2015 | 28739421 |
| SRR5657455 | Cow | Mexico | 2015 | 28739421 |
| SRR5657456 | Cow | Mexico | 2015 | 28739421 |
| SRR5657457 | Cow | Mexico | 2015 | 28739421 |
| SRR5657458 | Cow | Mexico | 2015 | 28739421 |
| SRR5657459 | Cow | Mexico | 2015 | 28739421 |
| SRR5657460 | Cow | Mexico | 2015 | 28739421 |
| SRR5657461 | Cow | Mexico | 2015 | 28739421 |
| SRR5657462 | Cow | Mexico | 2015 | 28739421 |
| SRR5657463 | Cow | Mexico | 2015 | 28739421 |
| SRR5657464 | Human | Mexico | 2013 | 28739421 |
| SRR5657465 | Human | Mexico | 2013 | 28739421 |
| SRR5657466 | Cow | Mexico | 2015 | 28739421 |
| SRR5657467 | Cow | Mexico | 2015 | 28739421 |
| SRR5657468 | Cow | Mexico | 2015 | 28739421 |
| SRR5657469 | Cow | Mexico | 2015 | 28739421 |
| SRR5657470 | Cow | Mexico | 2015 | 28739421 |
| SRR5657471 | Cow | Mexico | 2015 | 28739421 |
| SRR5657472 | Cow | Mexico | 2015 | 28739421 |
| SRR5657473 | Cow | Mexico | 2015 | 28739421 |
| SRR5657474 | Cow | Mexico | 2015 | 28739421 |
| SRR5657475 | Cow | Mexico | 2015 | 28739421 |
| SRR5657476 | Cow | Mexico | 2015 | 28739421 |
| SRR5657477 | Human | Mexico | 2012 | 28739421 |
| SRR5657478 | Human | Mexico | 2012 | 28739421 |
| SRR5657479 | Human | Mexico | 2012 | 28739421 |
| SRR5657480 | Human | Mexico | 2012 | 28739421 |
| SRR5657481 | Human | Mexico | 2012 | 28739421 |
| SRR5657482 | Human | Mexico | 2012 | 28739421 |
| SRR5657483 | Human | Mexico | 2012 | 28739421 |
| SRR5657484 | Human | Mexico | 2012 | 28739421 |
| SRR5657485 | Human | Mexico | 2012 | 28739421 |
| SRR5657486 | Human | Mexico | 2012 | 28739421 |
| SRR5657487 | Cow | Mexico | 2015 | 28739421 |
| SRR5657488 | Cow | Mexico | 2015 | 28739421 |
| SRR5657489 | Cow | Mexico | 2015 | 28739421 |
| SRR5657490 | Cow | Mexico | 2015 | 28739421 |
| SRR5657491 | Cow | Mexico | 2015 | 28739421 |
| SRR5657492 | Cow | Mexico | 2015 | 28739421 |
| SRR5657493 | Cow | Mexico | 2015 | 28739421 |
| SRR5657494 | Cow | Mexico | 2015 | 28739421 |
| SRR5657495 | Cow | Mexico | 2015 | 28739421 |
| SRR5657496 | Cow | Mexico | 2015 | 28739421 |
| SRR5657497 | Cow | Mexico | 2015 | 28739421 |
| SRR5657498 | Cow | Mexico | 2015 | 28739421 |
| SRR5657499 | Cow | Mexico | 2015 | 28739421 |
| SRR5657500 | Cow | Mexico | 2015 | 28739421 |
| SRR5657501 | Cow | Mexico | 2015 | 28739421 |
| SRR5657502 | Cow | Mexico | 2015 | 28739421 |
| SRR5657503 | Cow | Mexico | 2015 | 28739421 |
| SRR5657504 | Cow | Mexico | 2015 | 28739421 |
| SRR5657505 | Cow | Mexico | 2015 | 28739421 |
| SRR5657506 | Cow | Mexico | 2015 | 28739421 |
| SRR5657507 | Cow | Mexico | 2015 | 28739421 |
| SRR5657508 | Cow | Mexico | 2015 | 28739421 |
| SRR5657509 | Cow | Mexico | 2015 | 28739421 |
| SRR5657510 | Cow | Mexico | 2015 | 28739421 |
| SRR5657511 | Cow | Mexico | 2015 | 28739421 |
| SRR5657512 | Cow | Mexico | 2015 | 28739421 |
| SRR5657513 | Cow | Mexico | 2015 | 28739421 |
| SRR5657514 | Cow | Mexico | 2015 | 28739421 |
| SRR5657515 | Cow | Mexico | 2015 | 28739421 |
| SRR5657516 | Cow | Mexico | 2015 | 28739421 |
| SRR5657517 | Cow | Mexico | 2015 | 28739421 |
| SRR5657518 | Cow | Mexico | 2015 | 28739421 |
| SRR5657519 | Cow | Mexico | 2015 | 28739421 |
| SRR5657520 | Cow | Mexico | 2015 | 28739421 |
| SRR5657521 | Cow | Mexico | 2015 | 28739421 |
| SRR5657522 | Cow | Mexico | 2015 | 28739421 |
| SRR5657523 | Cow | Mexico | 2015 | 28739421 |
| SRR5657524 | Cow | Mexico | 2015 | 28739421 |
| SRR5657525 | Cow | Mexico | 2015 | 28739421 |
| SRR5657526 | Cow | Mexico | 2015 | 28739421 |
| SRR5657527 | Cow | Mexico | 2015 | 28739421 |
| SRR5657528 | Cow | Mexico | 2015 | 28739421 |
| SRR5657529 | Cow | Mexico | 2015 | 28739421 |
| SRR6705904 | Cow | Brazil | 2010 | 29259589 |
| SRR5430090 | Cow | Uruguay | 2015 | 29291727 |
| SRR5430091 | Cow | Uruguay | 2015 | 29291727 |
| SRR5430098 | Cow | Uruguay | 2014 | 29291727 |
| SRR5430569 | Cow | Uruguay | 2008 | 29291727 |
| SRR5430746 | Cow | Uruguay | 2014 | 29291727 |
| SRR5430747 | Cow | Uruguay | 2014 | 29291727 |
| SRR5430748 | Cow | Uruguay | 2015 | 29291727 |
| SRR5430749 | Cow | Uruguay | 2015 | 29291727 |
| SRR5430750 | Cow | Uruguay | 2014 | 29291727 |
| SRR5431711 | Cow | Uruguay | 2015 | 29291727 |
| SRR5431718 | Cow | Uruguay | 2015 | 29291727 |
| SRR5431719 | Cow | Uruguay | 1998 | 29291727 |
| SRR5431720 | Cow | Uruguay | 2013 | 29291727 |
| SRR5431721 | Cow | Uruguay | 2015 | 29291727 |
| SRR5431730 | Cow | Uruguay | 2015 | 29291727 |
| SRR5431732 | Cow | Uruguay | 2015 | 29291727 |
| SRR5431790 | Cow | Uruguay | 2015 | 29291727 |
| SRR5431791 | Cow | Uruguay | 2010 | 29291727 |
| SRR5431792 | Cow | Uruguay | 2008 | 29291727 |
| SRR5431793 | Cow | Uruguay | 2010 | 29291727 |
| SRR5431795 | Cow | Uruguay | 2005 | 29291727 |
| SRR6467885 | Cow | Canada | 2011 | 29650575 |
| SRR6467887 | Wood bison | Canada | 1985 | 29650575 |
| SRR6467889 | Cow | Canada | 2003 | 29650575 |
| SRR6467891 | Elk | Canada | 2006 | 29650575 |
| SRR6467893 | Cow | Canada | 2008 | 29650575 |
| SRR5486073 | Cow | Eritrea | 2014 | 29664901 |
| SRR5486074 | Cow | Eritrea | 2015 | 29664901 |
| SRR5486075 | Cow | Eritrea | 2015 | 29664901 |
| SRR5486076 | Cow | Eritrea | 2015 | 29664901 |
| SRR5486077 | Cow | Eritrea | 2015 | 29664901 |
| SRR5486078 | Cow | Eritrea | 2015 | 29664901 |
| SRR5486079 | Cow | Eritrea | 2015 | 29664901 |
| SRR5486080 | Cow | Eritrea | 2015 | 29664901 |
| SRR5486081 | Cow | Eritrea | 2015 | 29664901 |
| SRR5486082 | Cow | Eritrea | 2014 | 29664901 |
| SRR5486083 | Cow | Eritrea | 2014 | 29664901 |
| SRR5486084 | Cow | Eritrea | 2014 | 29664901 |
| SRR5486085 | Cow | Eritrea | 2014 | 29664901 |
| SRR5486086 | Cow | Eritrea | 2014 | 29664901 |
