# Supplementary material for: Molecular epidemiology and whole genome sequencing analysis of clinical Mycobacterium bovis from Ghana
Source: PLoS One. 2019 Mar 4;14(3):e0209395. doi: 10.1371/journal.pone.0209395 (PMC6398925; doi:10.1371/journal.pone.0209395)
Supplement: S2 Table — (DOCX) [file pone.0209395.s002.docx]

**Supplementary table S2: List of MTBC core genes for comparative mutational analysis**

| **Family of genes** | **List of genes** |
| --- | --- |
| Sulphur transporters | Rv2400c,Rv2398c,Rv2397c,Rv2399c,Rv1739c,Rv1707 |
| Phosphorus transporters | Rv0545c,Rv2281,Rv0932c,Rv0931c,Rv0934,Rv0933,Rv0828c,Rv0930,Rv0929,Rv0820 |
| Nitrogen transporters | Rv2920c,Rv0267,Rv0261c,Rv1737c,Rv2329c |
| Ion transporters | Rv1348,Rv1349,Rv2691,Rv2692,Rv1857,Rv1858,Rv1859,Rv0924c,Rv1239c,Rv2856,Rv1607,Rv2287,Rv3236c,Rv2877c,Rv0092,Rv0103c,Rv3270,Rv1469,Rv3200c,Rv3679,Rv3680,Rv3682,Rv0908,Rv1997,Rv1992c,Rv0425c,Rv0107c,Rv3743c,Rv0969,Rv1029,Rv1030,Rv1031,Rv1028A,Rv0143c,Rv0265c,Rv2025c,Rv3041c,Rv1811,Rv0362,Rv3044 |
| Carbohydrate transporters | Rv3331,Rv2833c,Rv2835c,Rv2832c,Rv2834c,Rv1236,Rv1237,Rv1238,Rv1235,Rv2037c,Rv2038c,Rv2039c,Rv2040c,Rv2041c,Rv2316,Rv2317,Rv2318,Rv1200,Rv2456c |
| Lipid transporters | Rv0402c,Rv0507,Rv0206c,Rv0450c,Rv0676c,Rv1557,Rv2942,Rv3823c,Rv2339,Rv1183,Rv0202c,Rv1522c,Rv1145,Rv1146 |
| Peptide transporters | Rv2585c,Rv1281c,Rv1280c,Rv1283c,Rv1282c,Rv3666c,Rv3665c,Rv3664c,Rv3663c |
| Amino acid transporters | Rv3759c,Rv3758c,Rv3757c,Rv3756c,Rv0072,Rv0073,Rv0048c,Rv1496,Rv1747,Rv1979c,Rv1986,Rv1999c,Rv2563,Rv3253c,Rv0522,Rv0899,Rv1704c,Rv0917,Rv2320c,Rv2564,Rv2127,Rv0346c |
| Cell invasion operon | Rv0169,Rv0170,Rv0171,Rv0172,Rv0174,Rv0173,Rv0175,Rv0176,Rv0177,Rv0178, Rv3414c |
| Lipid catabolism operon | Rv3545c,Rv3544c,Rv3543c,Rv3542c,Rv3541c,Rv3540c |
| Cholesterol catabolism operon | Rv3570c,Rv3569c,Rv3568c,Rv3567c |
| ESAT-6 secretory system 1 | Rv3864,Rv3865,Rv3866,Rv3867,Rv3868,Rv3869,Rv3870,Rv3871 |
| ESAT-6 secretory system 2 | Rv3874,Rv3875,Rv3880c,Rv3877,Rv3878 |
| Growth attenuation genes | Rv0557,Rv0469,Rv0930,Rv0862c,Rv1096,Rv1125,Rv1178,Rv1328,Rv1592c,Rv2112c,Rv2241,Rv2383c,Rv2737c,Rv3282,Rv3696c,Rv3409c,Rv0642c,Rv0470c,Rv2869c,Rv0186A,Rv2524c,Rv3499c,Rv3082c,Rv1242,Rv1246c,Rv1241,Rv1323,Rv1345,Rv1347c,Rv1350 |
| Polyketide synthase genes | Rv0405,Rv1013,Rv1180,Rv1181,Rv1527c,Rv1660,Rv1661,Rv1662,Rv1663,Rv1664,Rv1665,Rv2048c,MTB000057,Rv2946c,Rv2947c,Rv3800c,Rv3825c |
