# Supplementary material for: Molecular epidemiology and whole genome sequencing analysis of clinical Mycobacterium bovis from Ghana
Source: PLoS One. 2019 Mar 4;14(3):e0209395. doi: 10.1371/journal.pone.0209395 (PMC6398925; doi:10.1371/journal.pone.0209395)
Supplement: S3 Table — (DOCX) [file pone.0209395.s003.docx]

**Supplementary table S3: Mutations restricted to the 5 clinical *M. bovis* from Ghana relative to hMTBC**

| No | Gene | Common name | Family | Group | Mutation |
| --- | --- | --- | --- | --- | --- |
| 1 | *Rv0048c* | *Rv0048c* | amino acid transporters | cell wall and cell processes | G224S |
| 2 | *Rv0107c* | *ctpI* | ion transporters | cell wall and cell processes | A1327S |
| 3 | *Rv0107c* | *ctpI* | ion transporters | cell wall and cell processes | A136V |
| 4 | *Rv0107c* | *ctpI* | ion transporters | cell wall and cell processes | F1570fs |
| 5 | *Rv0169* | *mce1A* | transport and cell invasion operon | virulence, detoxification, adaptation | P359S |
| 6 | *Rv0402c* | *mmpL1* | lipid/fat transporters | cell wall and cell processes | L451P |
| 7 | *Rv0402c* | *mmpL1* | lipid/fat transporters | cell wall and cell processes | P568fs |
| 8 | *Rv0405* | *pks6* | polyketide synthase | lipid metabolism | A456fs |
| 9 | *Rv0820* | *phoT* | phosphorus transporters | cell wall and cell processes | F35L |
| 10 | *Rv0931c* | *pknD* | phosphorus transporters | regulatory | L276fs |
| 11 | *Rv0933* | *pstB* | phosphorus transporters | cell wall and cell processes | L64fs |
| 12 | *Rv1182* | *pks4* | polyketide synthase | lipid metabolism | D565A |
| 13 | *Rv1328* | *glgP* | growth attenuation | intermediary metabolism and respiration | D532G |
| 14 | *Rv1328* | *glgP* | growth attenuation | intermediary metabolism and respiration | V576F |
| 15 | *Rv1522c* | *mmpL12* | lipid_fat transporters | cell wall and cell processes | S947N |
| 16 | *Rv1527c* | *pks5* | polyketide synthase | lipid metabolism | F1439L |
| 17 | *Rv1607* | *chaA* | ion transporters | cell wall and cell processes | P6T |
| 18 | *Rv1661* | *pks7* | polyketide synthase | lipid metabolism | S1176P |
| 19 | *Rv1662* | *pks8* | polyketide synthase | lipid metabolism | A808V |
| 20 | *Rv1662* | *pks8* | polyketide synthase | lipid metabolism | D78Y |
| 21 | *Rv1662* | *pks8* | polyketide synthase | lipid metabolism | Y1469C |
| 22 | *Rv1707* | *N.A* | sulphur transporters | cell wall and cell processes | R438Q |
| 23 | *Rv1811* | *mgtC* | ion transporters | cell wall and cell processes | G187E |
| 24 | *Rv1979c* | *Rv1979c* | amino acid transporters | cell wall and cell processes | T35A |
| 25 | *Rv2127* | *ansP1* | amino acid transporters | cell wall and cell processes | G44S |
| 26 | *Rv2329c* | *narK1* | nitrogen transporters | cell wall and cell processes | S387A |
| 27 | *Rv2339* | *mmpL9* | lipid/fat transporters | cell wall and cell processes | A44V |
| 28 | *Rv2339* | *mmpL9* | lipid/fat transporters | cell wall and cell processes | W827trunc |
| 29 | *Rv2383c* | *mtbB* | growth attenuation | lipid metabolism | R263Q |
| 30 | *Rv2524c* | *fas* | growth attenuation | lipid metabolism | L400F |
| 31 | *Rv2946c* | *pks1* | polyketide synthase | lipid metabolism | A1360V |
| 32 | *Rv2946c* | *pks1* | polyketide synthase | lipid metabolism | V1283G |
| 33 | *Rv2955c* | *pks15* | conserved proteins | lipid metabolism | R27H |
| 34 | *Rv2956* | *N.A* | conserved proteins | information pathways | I237T |
| 35 | *Rv3082c* | *virS* | growth attenuation | virulence, detoxification, adaptation | R322C |
| 36 | *Rv3282* | *Rv3282* | growth attenuation | conserved hypothetical | A133S |
| 37 | *Rv3666c* | *dppA* | peptide transporters | cell wall and cell processes | E451G |
| 38 | *Rv3759c* | *proX* | amino acid transporters | virulence, detoxification, adaptation | A176V |
| 39 | *Rv3868* | *eccA1* | ESAT6 secretory system I | cell wall and cell processes | A243V |
| 40 | *Rv3868* | *eccA1* | ESAT6 secretory system I | cell wall and cell processes | A563T |
| 41 | *Rv3878* | *espJ* | ESAT6 secretory system II | cell wall and cell processes | E45K |
