# Supplementary material for: Molecular epidemiology and whole genome sequencing analysis of clinical Mycobacterium bovis from Ghana
Source: PLoS One. 2019 Mar 4;14(3):e0209395. doi: 10.1371/journal.pone.0209395 (PMC6398925; doi:10.1371/journal.pone.0209395)
Supplement: S4 Table — (DOCX) [file pone.0209395.s004.docx]

**Supplementary table S4: Distribution of *M. bovis*-restricted amino acid mutations among global collection of *M. bovis***

| Number | Gene | Common Name | Mutation | Present (n=772) | Present (%) |
| --- | --- | --- | --- | --- | --- |
| 1 | *Rv0048c* | *Rv0048c* | G224S | 770 | 99.74 |
| 2 | *Rv0107c* | *ctpI* | A1327S | 770 | 99.74 |
| 3 | *Rv0107c* | *ctpI* | A136V | 771 | 99.87 |
| 4 | *Rv0107c* | *ctpI* | F157fs | 769 | 99.61 |
| 5 | *Rv0169* | *mce1A* | P359S | 772 | 100.00 |
| 6 | *Rv0402c* | *mmpL1* | L451P | 772 | 100.00 |
| 7 | *Rv0402c* | *mmpL1* | P568fs | 772 | 100.00 |
| 8 | *Rv0405* | *pks6* | A456fs | 772 | 100.00 |
| 9 | *Rv0820* | *phoT* | F35L | 772 | 100.00 |
| 10 | *Rv0931c* | *pknD* | L376fs | 771 | 99.87 |
| 11 | *Rv0933* | *pstB* | L64fs | 772 | 100.00 |
| 12 | *Rv1181* | *pks4* | D505A | 768 | 99.48 |
| 13 | *Rv1328* | *glgP* | D532G | 772 | 100.00 |
| 14 | *Rv1328* | *glgP* | V576F | 771 | 99.87 |
| 15 | *Rv1522c* | *mmpL12* | S947N | 752 | 97.41 |
| 16 | *Rv1527c* | *pks5* | F1439L | 770 | 99.74 |
| 17 | *Rv1607* | *chaA* | P6T | 5 | 0.65 |
| 18 | *Rv1661* | *pks7* | S1176P | 740 | 95.85 |
| 19 | *Rv1662* | *pks8* | A808V | 756 | 97.93 |
| 20 | *Rv1662* | *pks8* | D78Y | 755 | 97.80 |
| 21 | *Rv1662* | *pks8* | Y1469C | 769 | 99.61 |
| 22 | *Rv1707* | *Rv1707* | R438Q | 760 | 98.45 |
| 23 | *Rv1811* | *mgtC* | G187E | 5 | 0.65 |
| 24 | *Rv1979c* | *Rv1979c* | T35A | 5 | 0.65 |
| 25 | *Rv2127* | *ansP1* | G44S | 771 | 99.87 |
| 26 | *Rv2329c* | *narK1* | S387A | 5 | 0.65 |
| 27 | *Rv2339* | *mmpL9* | A44V | 771 | 99.87 |
| 28 | *Rv2339* | *mmpL9* | W827trunc | 767 | 99.35 |
| 29 | *Rv2383c* | *mbtB* | R263Q | 769 | 99.61 |
| 30 | *Rv2524c* | *fas* | L400F | 5 | 0.65 |
| 31 | *Rv2946c* | *pks1* | A1360V | 766 | 99.22 |
| 32 | *Rv2946c* | *pks1* | V1283G | 764 | 98.96 |
| 33 | *Rv2955c* | *Rv2955c* | R27H | 772 | 100.00 |
| 34 | *Rv2956* | *Rv2956* | I237T | 769 | 99.61 |
| 35 | *Rv3082c* | *virS* | R322C | 770 | 99.74 |
| 36 | *Rv3282* | *Rv3282* | A133S | 770 | 99.74 |
| 37 | *Rv3666c* | *dppA* | E451G | 755 | 97.80 |
| 38 | *Rv3759c* | *proX* | A176V | 770 | 99.74 |
| 39 | *Rv3868* | *eccA1* | A243V | 768 | 99.48 |
| 40 | *Rv3868* | *eccA1* | A563T | 5 | 0.65 |
| 41 | *Rv3878* | *espJ* | E45K | 771 | 99.87 |
