# Supplementary figures and images for: Molecular epidemiology and whole genome sequencing analysis of clinical Mycobacterium bovis from Ghana
Source: PLoS One. 2019 Mar 4;14(3):e0209395. doi: 10.1371/journal.pone.0209395 (PMC6398925; doi:10.1371/journal.pone.0209395)

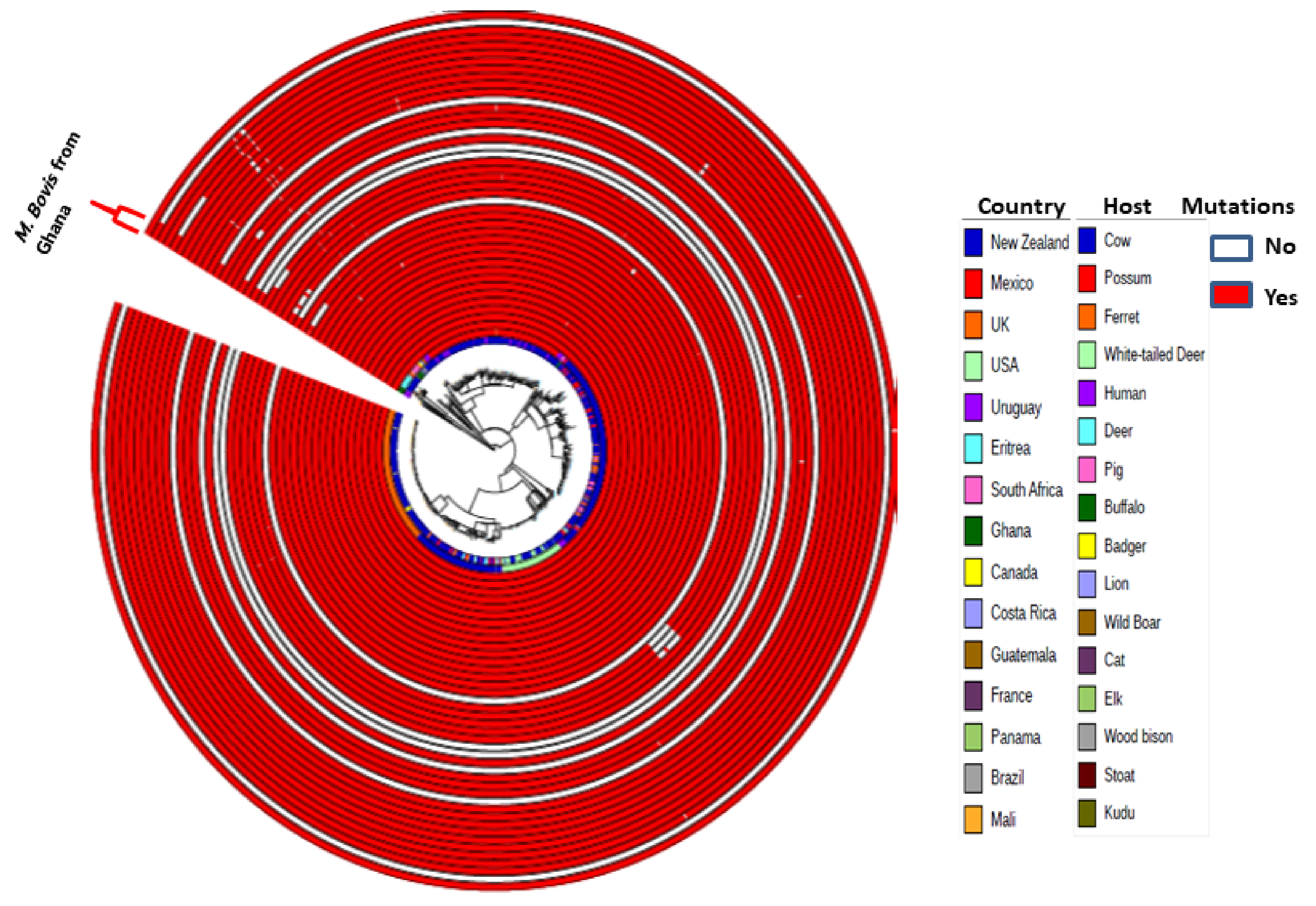

Supplement: S1 Fig — Distribution of the M. bovis restricted amino acid mutations on the midpoint-rooted maximum-likelihood phylogeny of 772 global collection of M. bovis genomes. Mutation present and absent are represented by the red and white blocks respectively. (TIF) [file pone.0209395.s005.tif]
